# Low serum vitamin B_12_ levels are associated with degenerative rotator cuff tear

**DOI:** 10.1186/s12891-021-04231-7

**Published:** 2021-04-17

**Authors:** Jae Hwa Kim, Go-Tak Kim, Siyeoung Yoon, Hyun Il Lee, Kyung Rae Ko, Sang-Cheol Lee, Do Kyung Kim, Jaeyeon Shin, So-young Lee, Soonchul Lee

**Affiliations:** 1 Department of Orthopaedic Surgery, CHA Bundang Medical Center, CHA University School of Medicine, 335 Pangyo-ro, Bundang-gu, Seongnam-si, Gyeonggi-do 13488 Republic of Korea; 2 Department of Orthopedic Surgery, Ilsan Paik Hospital, Inje University, Goyang-si, Gyeonggi-do 10380 Republic of Korea; 3 Department of Orthopaedic Surgery, Samsung Medical Center, Sungkyunkwan University, School of Medicine, 81 Irwon-ro, Gangnam-gu, Seoul, 06351 Republic of Korea; 4 CHA Graduate School of Medicine, 120 Hyeryong-ro, Pocheon, 11160 Republic of Korea; 5 Department of Computer Science, College of IT Engineering, SeMyung University, Semyung-ro, Jecheon-si, Chung-cheong bukdo 27136 South Korea; 6 Department of Internal Medicine, CHA Bundang Medical Center, CHA University School of Medicine, 335 Pangyo-ro, Bundang-gu, Seongnam-si, Gyeonggi-do 13488 Republic of Korea

**Keywords:** Vitamin B_12_, Rotator cuff, Degenerative, Tear

## Abstract

**Background:**

Vitamin B_12_ (Vit B_12_) deficiency results in elevated homocysteine levels and interference with collagen cross-linking, which may affect tendon integrity. The purpose of this study was to investigate whether serum Vit B_12_ levels were correlated with degenerative rotator cuff (RC) tear.

**Methods:**

Eighty-seven consecutive patients with or without degenerative RC tear were enrolled as study participants. Possible risk factors (age, sex, medical history, bone mineral density, and serum chemistries including glucose, magnesium, calcium, phosphorus, zinc, homocysteine, Vitamin D, Vit B_12_, homocysteine, and folate) were assessed. Significant variables were selected based on the results of univariate analyses, and a logistic regression model (backward elimination) was constructed to predict the presence of degenerative RC tear.

**Results:**

In the univariate analysis, the group of patients with degenerative RC tear had a mean concentration of 528.4 pg/mL Vit B_12_, which was significantly lower than the healthy control group (627.1 pg/mL). Logistic regression analysis using Vit B_12_ as an independent variable revealed that Vit B_12_ concentrations were significantly correlated with degenerative RC tear (*p* = 0.044). However, Vit B_12_ levels were not associated with tear size.

**Conclusion:**

Low serum levels of Vit B_12_ were independently related to degenerative RC tear. Further investigations are warranted to determine if Vit B_12_ supplementation can decrease the risk of this condition.

**Supplementary Information:**

The online version contains supplementary material available at 10.1186/s12891-021-04231-7.

## Background

Degenerative rotator cuff (RC) tear typically affects the supraspinatus tendon. This relatively common shoulder lesion can cause significant pain and disability. The number of affected patients is expected to increase as the average life expectancy increases. Approximately 40% of the US population > 60 years of age is affected by this condition annually, and 30,000 to 75,000 RC repairs are performed each year [1]. If untreated, the size of the tear may increase over time. Surgical repair is commonly performed in patients with persistent symptoms [2]. In the USA, the use of the RC repair procedure has increased considerably since 2000, and it is one of the most frequently performed orthopedic surgical procedures in the country [3]. Repair of degenerative RC tears has increased two-fold since 2010 [2, 4].

Study results suggest that the pathogenesis and biochemical changes associated with degenerative RC tear arise from a combination of extrinsic impingement and intrinsic alterations, including increased oxidative stress, neurogenic dysregulation, decreased blood flow, and changes in the extracellular matrix within tendon tissue [5, 6]. Histopathologically, tendon changes include collagen fiber thinning and disorganization, development of granulation tissue, glycosaminoglycan infiltration, fibrocartilaginous metaplasia, calcification, fatty degeneration, and necrosis of the tendon margin with cell apoptosis [7]. These changes are also present in macroscopically intact tendons during the early stages of the degenerative process [7].

Two steps are required to absorb the essential water-soluble vitamin B_12_ (Vit B_12_) (cobalamin) from food. First, in the stomach, Vit B_12_ bound to proteins in food is separated by hydrochloric acid. Vit B_12_ then combines with gastric intrinsic factor protein and is absorbed by the body. Vit B_12_ is obtained via ingestion of sources including meat, fish, dairy products, fortified cereals, and supplements [8]. It is crucial for the maintenance of neuronal health and hematopoiesis [9]. Because Vit B_12_ has antioxidant properties, a deficiency contributes to oxidative stress and the onset of age-related diseases [10]. However, the clinical relationships between Vit B_12_ deficiency and degenerative diseases remain unclear. Induction of nutrient reallocation to processes necessary for survival can occur during asymptomatic subclinical micronutrient deficiencies [11]. Over the long term, these oxidative stress and other factors contribute to the development of age-related diseases that result from neglect of daily metabolic processes [12].

Studies have not clearly revealed the relationships between Vit B12 levels and degenerative RC tear to the best of our knowledge. The purpose of this study was to evaluate whether serum Vit B_12_ levels were independently associated with the occurrence of degenerative RC tear.

## Methods

### Ethics statement

The study protocol (No. 2016–11–009-019) was approved by the institutional review board of this institute. Study procedures were performed following Declaration of Helsinki principles. Written informed consent was obtained from each participant.

### Study design and population

In this prospective study, participants were enrolled at a hospital in South Korea between January 2018 and June 2019. Only participants 55–80 years of age were included in the study. Exclusion criteria were patients with a history of malignancy, active infection, auto-immune disease, or previous fracture or surgery of structures in or around the shoulder. No patient had megaloblastic anemia or neuropathy associated with clinically established Vit B_12_ deficiency.

The study group was divided into the RC tear group and the non-RC tear group. For inclusion in the RC tear group, a patient was required to have a full-thickness RC tear involving the supraspinatus or other RC tendons, or both, and admission to the study institute for arthroscopic RC repair. A non-operative treatment protocol was followed for at least 3 months before surgery. The non-operative treatment protocol included stopping offending activities, physiotherapy (manipulation, stretching, ultrasound, electrical stimulation, and strengthening exercises), corticosteroid injections, anti-inflammatory agents, and extracorporeal shock wave therapy. Only patients with degenerative RC tear were included in the RC group. The condition was confirmed by detailed history taking including the significant trauma history around the shoulder with a physical examination and magnetic resonance imaging (MRI) (Signa Architect 3.0 T; General Electronic Healthcare) to exclude the traumatic RC tear. MRI was used to measure tear size (width and length). During the study period, 75 patients had a diagnosis of full-thickness RC tear and underwent surgical treatment. Fourteen of these patients refused to participate in the study. Thirteen patients were excluded from the study were because the data were incomplete. One patient was excluded because of auto-immune disease (rheumatoid arthritis), malignancy (2 patients), or a history of fracture (3 patients) or surgery in the affected shoulder (2 patients). A total of 40 patients were assigned to the RC tear group.

**Fig. 1:**
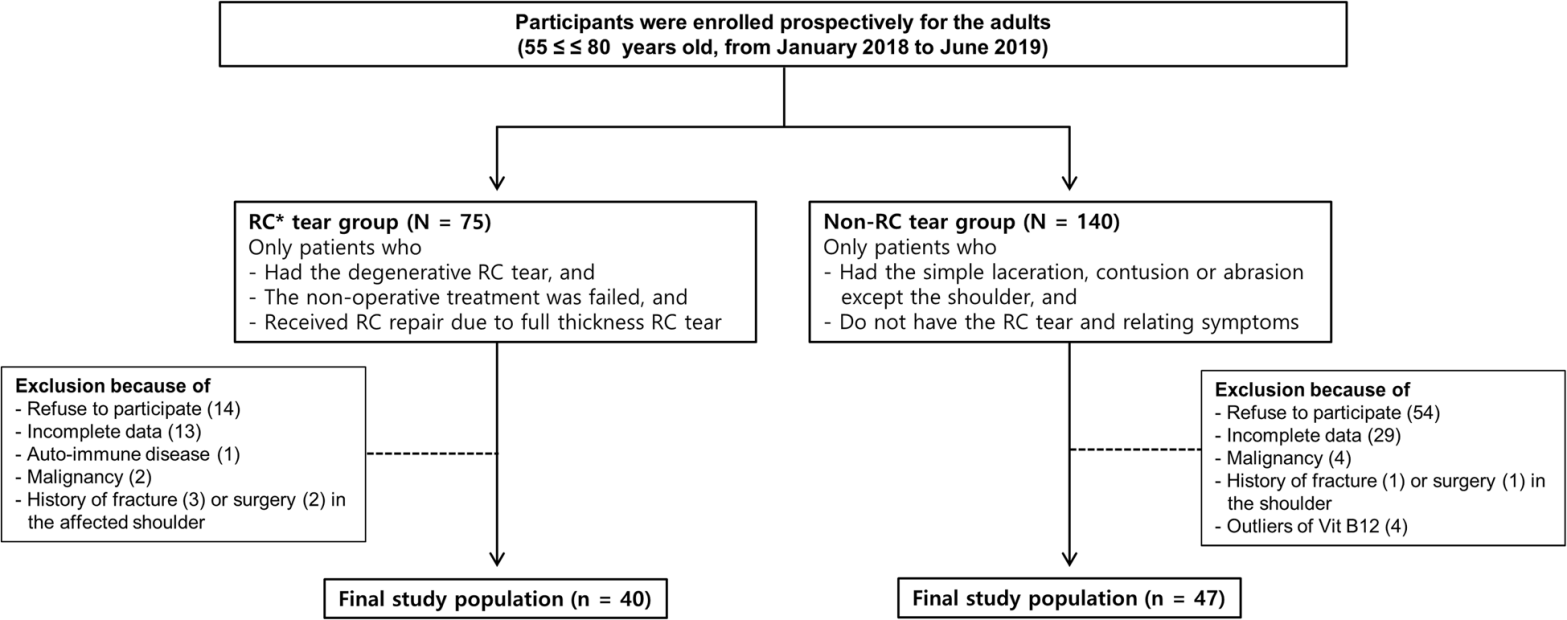
Flow diagram of results after inclusion and exclusion criteria were applied in this study. ^*^Rotator cuff

To be eligible for inclusion in the non-RC tear group, a patient had no RC tear or associated symptoms or clinical signs (e.g., loss of shoulder motion, signs of impingement, tenderness), and visited the institute’s orthopedic clinic. Eligible patients visited the orthopedic clinic during the study period because of minor trauma (dressing superficial lacerations, contusions, and abrasions) of any area except the shoulder region. None previously underwent surgery relating to RC tear. A total of 47 healthy participants who met these criteria and agreed to participate in the study were assigned to the non-RC tear group (Fig. 1).

### Variables and data collection

General information collected for each participant included demographic data and medical history. Data on biochemical markers including glucose, magnesium (Mg), calcium (Ca), phosphorus (P), zinc (Zn), homocysteine (HCY), vitamin D (Vit D), folate, and Vit B_12_ were evaluated. To measure biochemical markers, approximately 10-ml venous blood samples were collected in vacuum-sealed blood tubes in the outpatient or inpatient clinic after an overnight fast. Each sample was centrifuged (3000 rpm, 15 min) and then stored at − 80 °C. Serum electrolyte levels were obtained using a Roche COBAS E701 electrolyte analyzer. Serum Vit B_12_ was determined via electrochemiluminescence immune assay (ELISA) (Roche Elecsys 2010 analyzer; Switzerland). The remaining components were analyzed using a Siemens Atellica auto-analyzer. All samples were analyzed at the hospital’s laboratory services in the department of clinical pathology at our institution. All staff who performed the analyses were blinded to the study group assignments [13].

Bone mineral density (BMD, g/cm^2^) was also measured because study findings indicate that BMD is associated with RC tear development [14]. Dual-energy X-ray absorptiometry (Hologic QDR-4500, USA; software version 9.30.044) was used to measure lumbar spine (L1 - L4) and proximal femur (femur neck and total hip) areal BMD.

### Arthroscopic examination and RC repair

The same surgeon performed each arthroscopic procedure with the patient in the lateral decubitus position. After the surgeon accessed the subacromial space, partial bursectomy and debridement were used to visualize torn RC tendon margins. When the torn cuff was visible, the RC repairs were performed based on tear size [15].

### Sample size calculation and statistical analyses

The results were presented as mean (± standard deviation) values within groups. Variables with *P*-values < 0.05 were considered statistically significant. The minimum sample size was calculated to ensure sufficient power to analyze the primary outcome variable (RC tear). The parameters included in the sample size estimate were: two-sided t-test, alpha = 0.05, power = 0.80, assumed difference in Vit B_12_ levels of 21.2% between non-RC tear (744 ± 239 pg/ml) and RC tear (587 ± 215 pg/ml) groups. The values used were based on results of preliminary studies. The calculated minimum sample size was 34 for each group.

To identify correlations between nominal variables and RC tear, Chi-square tests were performed between the RC tear group and non-RC tear group for diabetes mellitus, hypertension, and stroke history. Independent sample t-tests were performed for continuous variables. Continuous variables included glucose, Mg, Ca, P, Zn, Vit D, Vit B_12_, folate, HCY and BMD.

Logistic regression analysis (backward elimination) was performed to identify independent predictors of RC tear while adjusting for demographic and clinical variables. The variables were retained or deleted based on their statistical contribution. After this process, we were able to find out following 8 variables: Sex, Age, DM, Glucose, P, Vit D, Vit B_12,_ and BMD.

A bivariate analysis was done to compare Vit B_12_ by RC tear using log-binomial regression to calculate relative risk. We split the Vit B_12_ according to the quartiles into 4 groups: samples divided by the cut-off point 25th (460.1 pg/mL), 50th (559.0 pg/mL), and 75th (695.5 pg/mL) percentile. Unadjusted and adjusted relative risks and 95% confidence intervals were reported. Adjusted analyses were controlled for eight variables selected by backward elimination regression previously.

Analysis of variance (ANOVA) was used for the analysis of differences in Vit B_12_ concentrations according to the ranges of retraction (length) and anteroposterior extension (width). These statistical analyses were performed using G*Power software and R version 3.5.2 for Windows.

## Results

The mean ages of the patients in the non-RC tear and RC tear groups were 59.3 ± 5.9 years and 61.0 ± 5.3 years, respectively, which was not statistically significant. However, the male had the 1.35 times higher chance of RC tear compared to the female (57.5% vs 42.5%), which was statistically significant (*P* = 0.017). Among the biochemical markers, the non-RC tear group had the significantly higher serum Vit B_12_ levels than the RC tear group (528.4 ± 145.7 pg/mL for the RC tear vs 627.1 ± 183.0 pg/mL for the non-RC tear group) (*P* = 0.007). The mean Vit D level was also lower in the RC tear group as 15.7 ± 7.2 ng/mL compared to the non-RC tear group as 21.6 ± 10.0 ng/mL with the statistical significance (*P* = 0.002). Next, the mean P level was significantly different between RC tear and non-RC tear group as 3.2 ± 0.6 mg/dL and 3.6 ± 0.7 mg/dL, respectively (*P* = 0.008). The other parameters showed no significant relationships with RC tear in the univariate analyses. Table 1 presents results for the baseline clinical and biochemical characteristics of each group.

**Table 1:** Demographic characteristics of all variables, non-RC tear group versus RC tear group

	RC tear (*n* = 40)	Non-RC tear (*n* = 47)	*P*-value
Sex			0.017**
Male	23 (57.5%)	14 (29.8%)	
Female	17 (42.5%)	33 (70.2%)	
Age (years)	61.0 (5.3)	59.3 (5.9)	0.142*
DM			0.970**
Yes	7 (17.5%)	7 (14.9%)	
No	33 (82.5%)	40 (85.1%)	
HTN			0.823**
Yes	12 (30.0%)	12 (25.5%)	
No	28 (70.0%)	35 (74.5%)	
CVA			0.887**
Yes	2 (5.0%)	1 (2.1%)	
No	38 (95.0%)	46 (97.9%)	
Glucose (mg/dL)	109.0 (21.5)	117.7 (34.8)	0.159*
Mg (mg/dL)	2.0 (0.3)	2.0 (0.2)	0.277*
Ca (mg/dL)	9.2 (0.4)	9.2 (0.7)	0.862*
P (mg/dL)	3.2 (0.6)	3.6 (0.7)	0.008*
Zn (mg/dL)	74.8 (12.8)	71.3 (12.6)	0.203*
HCY (μmol/L)	9.4 (2.4)	9.3 (3.1)	0.924*
Vit D (ng/mL)	15.7 (7.2)	21.6 (10.0)	0.002*
Vit B_12_ (pg/mL)	528.4 (145.7)	627.1 (183.0)	0.007*
Folate (ng/mL)	8.6 (6.5)	8.2 (4.4)	0.739*
BMD (g/cm^2^)	−1.8 (1.2)	−1.4 (1.2)	0.116*

Results are presented as mean (standard deviations) values. *RC* Rotator cuff, *DM* Diabetes mellitus, *HTN* Hypertension, *CVA* Cerebrovascular attack, Mg: Magnesium, *Ca* Calcium, *P* Phosphate, *Zn* Zinc, *HCY* Homocysteine, *Vit D* Vitamin D, *Vit B*_*12*_ Vitamin B_12_, *BMD* Bone mineral density. *: T-test, **: Pearson chi-square test

After controlling for confounding variables in logistic regression (backward elimination), the following variables were independently associated with RC tear; Sex, DM, Age, Glucose, Vit D, and Vit B_12_ (Table 2). Sex was more likely (odds ratio [OR] = 7.96; 95% confidence interval [CI] = 1.9–41.57; *P* = 0.008) to receive a significant variable among RC tear group and non-RC tear group. Age was positively associated with RC tear, after adjustment for other factors (OR = 1.18; 95% CI = 1.05–1.36; *P* = 0.009). DM was associated with RC tear (OR = 8.74; 95% CI = 1.24–76.79; *P* = 0.035). Glucose (OR = 0.97; 95% CI = 0.93–0.99; *P* = 0.031), Vit D (OR = 0.89; 95% CI = 0.82–0.96; *P* = 0.006) were negatively associated with RC tear. The analysis revealed that after adjustment for other factors, serum Vit B_12_ level was negatively associated with RC tear (OR = 0.99; 95% CI = 0.99–1; *P* = 0.044).

**Table 2:** Multivariate analysis: Logistic regression with backward elimination selection AIC

	First step AIC = 102.03			Last step AIC = 92.344		
	OR	95% CI	*P*-value	OR	95% CI	*P*-value
Sex	10.26	1.88–76.12	0.012	7.96	1.9–41.57	0.008
Age (years)	1.19	1.05–1.38	0.013	1.18	1.05–1.36	0.009
DM	7.74	1–71.65	0.054	8.74	1.24–76.79	0.035
HTN	0.9	0.21–3.66	0.887			
CVA	0.37	0.01–30.94	0.633			
Glucose (mg/dL)	0.97	0.94–1	0.107	0.97	0.93–0.99	0.031
Mg (mg/dL)	0.3	0.01–7.03	0.484			
Ca (mg/dL)	0.67	0.22–1.96	0.468			
P (mg/dL)	0.38	0.11–1.08	0.091	0.39	0.12–1.01	0.073
Zn (mg/dL)	1.02	0.96–1.09	0.526			
HCY (μmol/L)	0.88	0.63–1.18	0.433			
Vit D (ng/mL)	0.89	0.8–0.97	0.011	0.89	0.82–0.96	0.006
Vit B_12_ (pg/mL)	0.99	0.99–1	0.026	0.99	0.99–1	0.044
Folate (ng/mL)	1.06	0.94–1.21	0.384			
BMD (g/cm^2^)	0.6	0.3–1.14	0.129	0.61	0.33–1.08	0.101

*AIC* Akaike Information Criterion, *OR* Odds ratio, *SE* Standard error, *CI* Confidence interval

Next, low Vit B_1_2 group had the higher RR for the RC tear in general. In comparison to the ‘Vit B_12_ < 460.1 pg/mL’ group, the ‘559 - 695.5’ and ‘695.5 -’ group were less likely to occur degenerative RC tear (RR = 0.73, CI = 0.34–1.45 and RR = 0.64, CI = 0.28–1.31). While the 460.1–559.0 group was more likely to occur degenerative RC tear (RR = 0.73, CI = 0.34–1.45) (Table 3).

**Table 3:** Relative risks of Vit B_12_ group according to RC tear

Variable	Total (n)	Outcome n (%)	Crude RR	95% CI	Adjusted RR^a^	95% CI
Vit B_12_ (pg/mL)						
460.1	22	11 (50%)	1	Reference	1	Reference
460.1–559	21	14 (66.7%)	0.75	0.42–1.3	0.92	0.57–1.49
559–695.5	22	8 (36.4%)	1.37	0.69–2.9	1.09	0.66–1.8
695.5 -	23	7 (30.4%)	1.57	0.76–3.6	1.05	0.62–1.77

^a^Adjusted for Sex, Age, DM, Glucose, P, Vit D, BMD; *RR* Relative risk with a log-binomial regression, *CI* Confidence interval

We also performed an ANOVA to determine whether there was a correlation between RC tear size and Vit B_12_ level in the RC tear group only. The results indicated that there was no significant difference in Vit B_12_ level and MRI-measured RC tear size. These results are presented in Supplemental Table 1.

## Discussion

This study was designed to examine whether there was an association between serum Vit B_12_ levels and degenerative RC tear in adults 55 to 80 years of age. Factors found to affect degenerative RC tear include age, diabetes, hyperlipidemia, and Vit D deficiency [16]. Few studies have reported results on the relationship between Vit B_12_ and RC tear. We measured Vit B_12_ levels prospectively in RC tear and non-RC tear patients and found that patients with lower Vit B_12_ levels had greater odds of having a degenerative RC tear.

Hashimoto et al. [17] reported age-related histological changes including collagen fiber thinning, disorientation, and fatty infiltration in the RC tear. Tendon properties and functions are causally related to the architecture and quality of collagen fibers. Collagen is the primary extracellular matrix of RC tendons, and the major focus of tenocyte metabolism is to maintain matrisome integrity by regulating collagen [18].

The pathogenesis of degenerative RC tear remains incompletely understood but can include impaired collagen synthesis, oxidative stress, chronic inflammation, ischemia, and impaired healing [19]. Tendinopathy pathogenesis may include decreased tendon cell collagen synthesis and increased collagen degradation in the tendon matrix [20]. Due to age-dependent reductions in tendon cells and enzymes essential for collagen synthesis, collagen synthesis declines with age. Delayed repair of soft tissues such as tendons is also associated with old age [21, 22]. These changes create a more fragile injury-prone area. Individuals who perform repetitive tasks during daily activities, jobs, or sports are at greater risk of these changes [23].

Some previous studies that reported the roles of Vit B_12_ in collagen production and cross-linking included consideration of HCY. Vit B_12_ is an essential HCY degrading remethylation and trans sulfuratin pathways co-enzyme [11]. Therefore, Vit B_12_ deficiency is a main cause of elevated HCY serum concentrations. Considering hyperhomocysteinemia and collagen, Lee et al. [24] investigated the effect of HCY on the production of matrix metalloproteinase (MMP). They concluded that increased HCY levels are associated with increased MMP production, which is implicated in collagen cross-linking breakdown and related to poor healing of RC tendon tears [25]. Robert et al. [26] found that a high serum HCY concentration may weaken bone by interfering with collagen cross-linking. Our findings also suggested that Vit B_12_ has a specific role that affects RC tendon structural properties via the pathways mentioned above.

Oxidative stress has an important role in tendinopathy development [27, 28]. Reactive oxygen species (ROS) modulate physiological events (e.g., tenocyte proliferation and differentiation, inflammation, and healing) [29]. Cell injury, senescence, and death can be caused by extensive ROS exposure or insufficient cellular antioxidant capacity, or both [30]. Vit B_12_ might be implicated in the pathogenesis of tendinopathy via modulation of oxidative stress. Potential Vit B_12_ antioxidant properties include: (1) direct ROS (e.g., superoxide) scavenging; (2) indirect ROS scavenging stimulation via glutathione preservation; (3) cytokine and growth factor production modulation for protection from oxidative stress induced by the immune response; (4) reduction of homocysteine-induced oxidative stress; and (5) reduction of oxidative stress caused by advanced glycation end products [10]. Tomohiro et al. [31] *also found* reductions in cellular *L*-ascorbic acid levels. During Vit B_12_ deficiency, this potent antioxidant participates in collagen biosynthesis in most mammals. They found that reductions resulted in concurrent disordered biosynthesis of worm collagen [32].

Cytokines regulate host immune responses to inflammation, infection, and trauma. These molecular messengers participate in matrix turnover during tendinopathy, tenocyte activity, and tendon matrix gene expression [33]. Among the various cytokines, up-regulation of pro-inflammatory cytokines is associated with RC tendinopathy in rat and human models [34]. Al-Daghri et al. [35] measured systemic Vit B_12_ concentrations with pro-inflammatory cytokines and concluded that serum Vit B_12_ concentrations were associated with pro-inflammatory cytokines. In their in vitro study, Jinous et al. [36] found that low adipocyte Vit B_12_ levels induced greater gene expression and secretion of pro-inflammatory cytokines. Their results suggest that this change results in adipocyte dysfunction.

Regarding the diagnostic criteria for Vit B_12_ deficiency, the normal range is classically defined as 200–900 (or 700) pg/mL [13]. Using these criteria, all except two participants had levels within the normal range. Some authors suggest even lower levels (e.g., 100 pg/mL) as the lower limit of the normal range [37]. However, this normal range was defined based on the development of anemia or neurological disorders and treatment using Vit B_12_ supplementation. Green and Hannibal et al. suggested that subclinical Vit B_12_ levels are 119–200 pmol/L (161.28–271.07 pg/mL) serum Vit B_12_ [9, 38]. However, while an individual remains apparently symptom-free, these subclinical levels can induce long-term damage to macromolecules (e.g., nucleic acids, proteins, and lipids) [39]. In this context, Mitsuyama et al. proposed the optimal range of Vit B_12_ as 500–1300 pg/mL although the classical normal range is much lower [40]. Likewise, other experts suggested that the optimal range for HCY is < 7 μmol/L for good health although the classical standard reference range was 5.0–12 μmol/L and these optimal range had a significant association with a lower likelihood of stroke, atherosclerosis, and improved overall cardiovascular function [41]. Collectively, the classical normal range might not be applicable in this study because the relationships between Vit B_12_ and other aging-related diseases such as degenerative tendinopathy were not considered.

This study had some limitations. First, we did not examine whether Vit B_12_ supplementation improved clinical results. Research is needed to investigate the effects of Vit B_12_ supplementation on healing after an RC tear. Second, as discussed above, Vit B_12_ deficiency is closely related to higher HCY levels. Still, although it was higher in the non-RC tear group, serum HCY levels were not significantly different between the RC tear and non-RC tear groups (*P* = 0.774, Table 1). We speculate that the sample size was insufficient to detect a statistically significant difference. Long-term follow-up studies with larger groups of patients and focusing on HCY are needed. Last, we measured Vit B_12_ only one time. To analyze the long-term effects of low serum Vit B_12_ levels, serial follow-up measurements of Vit B_12_ levels should be performed.

## Conclusions

In conclusion, few studies about the relationship between Vit B_12_ and degenerative tendinopathy have been reported to date. Our results suggested that Vit B_12_ deficiency is an independent risk factor for RC tear. Further investigations are needed to understand relationships between the effects of Vit B_12_ and its byproducts on the structural properties of the RC tendon.

## Supplementary Information

**Additional file 1: Table S1.** Correlation between serum Vit B_12_ level and RC tear size measured by MRI.

## Data Availability

The datasets generated during and analyzed during the current study are not available due to privacy or ethical restrictions but are available from the corresponding author on reasonable request.

## Abbreviations

Vit B_12_: Vitamin B_12_
RC: Rotator cuff
MRI: Magnetic resonance imaging
Mg: Magnesium
Ca: Calcium
P: Phosphorus
Zn: Zinc
HCY: Homocysteine
Vit D: Vitamin D
ELISA: Electrochemiluminescence immune assay
BMD: Bone mineral density
ANOVA: Analysis of variance
OR: Odds ratio
MMP: Matrix metalloproteinase
ROS: Reactive oxygen species
