# Supplementary material for: Low serum vitamin B12 levels are associated with degenerative rotator cuff tear
Source: BMC Musculoskelet Disord. 2021 Apr 17;22:364. doi: 10.1186/s12891-021-04231-7 (PMC8053277; doi:10.1186/s12891-021-04231-7)
Supplement: Supplementary file 1 — Additional file 1: Table S1. Correlation between serum Vit B12 level and RC tear size measured by MRI. [file 12891_2021_4231_MOESM1_ESM.docx]

**Supplemental table 1. Correlation between serum Vit B_12_ level and RC tear size measured by MRI**

|  |  | **Retraction size** |
| --- | --- | --- |
| **Vit B_12_** | Pearson’s R | -0.09 |
|  | P-value | 0.5781 |
|  | 95% CI Upper | 0.227 |
|  | 95% CI Lower | -0.391 |

MRI: Magnetic resonance image, RC: Rotator cuff, Vit B_12_: Vitamin B_12_, CI: Confidence interval
